# LncRNAs in oncogenic microenvironment: from threat to therapy

**DOI:** 10.3389/fcell.2024.1423279

**Published:** 2025-03-13

**Authors:** Dipanjan Roy, Bireswar Bhattacharya, Rudra Chakravarti, Prabhjot Singh, Mansi Arya, Anirban Kundu, Ajay Patil, Bhukiya Siva, Sunny Mehta, Tawsif Ahmed Kazi, Dipanjan Ghosh

**Affiliations:** Department of Natural Products, National Institute of Pharmaceutical Education and Research-Kolkata, Kolkata, India

**Keywords:** lncRNA, cancer, tumor microenvironment, cancer therapy, inflammation, apoptosis

## Abstract

LncRNAs are RNA molecules of more than 200 nucleotides in length and participate in cellular metabolism and cellular responses through their diverse interactomedespite having no protein-coding capabilities. Such significant interactions also implicate the presence of lncRNAs in complex pathobiological pathways of various diseases, affecting cellular survival by modulating autophagy, inflammation and apoptosis. Proliferating cells harbour a complex microenvironment that mainly stimulate growth-specific activities such as DNA replication, repair, and protein synthesis. They also recognise damages at the macromolecular level, preventing them from reaching the next-generation. LncRNAs have shown significant association with the events occurring towards proliferation, regulating key events in dividing cells, and dysregulation of lncRNA transcriptome affects normal cellular life-cycle, promoting the development of cancer. Furthermore, lncRNAs also demonstrated an association with cancer growth and progression by regulating key pathways governing cell growth, epithelial-mesenchymal transition and metastasis. This makes lncRNAs an attractive target for the treatment of cancer and can also be used as a marker for the diagnosis and prognosis of diseases due to their differential expression in diseased samples. This review delves into the correlation of the lncRNA transcriptome with the fundamental cellular signalling and how this crosstalk shapes the complexity of the oncogenic microhabitat.

## 1 Introduction

**FIGURE 1:**
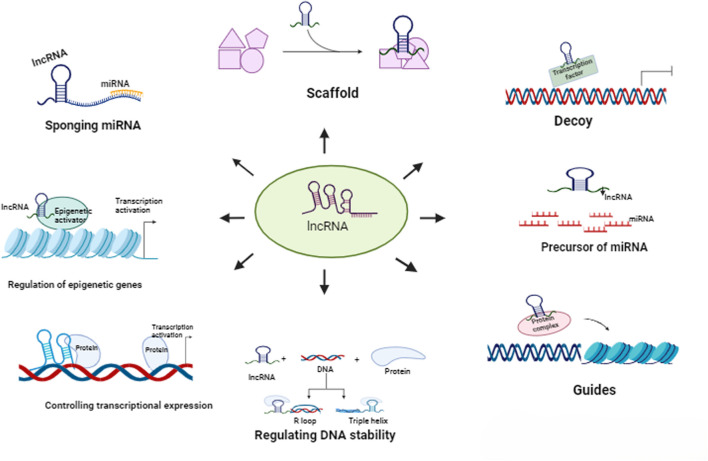
Functions of lncRNAs

RNA can be considered as the transient stage of gene expression. Genetic information in the DNA of an individual gets transcribed as RNA, and RNA is then translated into protein by the translation machinery. However, not all RNA molecules are destined to be protein encoders. Non-coding RNA molecules that have no protein products, such as tRNAs, rRNAs, miRNAs, siRNAs, hnRNAs, etc., are also expressed in cells. Earlier, these molecules were considered to play a limited role in the growth and development of organisms. But these all changed when studies found the involvement of such RNAs in various avenues of cellular importance, such as chromatin remodelling, gene expression, gene silencing, RNA interference, regulation of protein function, etc. Long non-coding RNAs or lncRNAs are a collective of untranslated RNAs having a length of more than 200 nucleotides. Despite not being encoded into proteins, their expression is highly regulated in mammalian cells via histone (epigenetic) methylation, transcription factors, promoters and even by other non-coding RNAs like miRNAs (Wu et al., 2014). lncRNAs can be classified based on several criteria such as length (Long non-coding RNAs, Large non-coding RNAs, Very large non-coding RNAs, etc.), location of the RNA respective to protein-coding genes (Intergenic, Antisense, Bidirectional, Intronic), location of the RNA respective to regulatory elements (Pseudogenes, Telomeres, Centromeres, Promoter-associated ncRNAs, etc.), lncRNA biogenesis pathway (Stable unannotated transcripts, Cryptic unstable transcripts, Meiotic unannotated transcripts, etc.), subcellular localisation of the RNA (Cytoplasmic lncRNAs, Nuclear lncRNAs, Mitochondrial lncRNAs), function (Scaffolds, Guides, Decoys, Precursors) and association to biological processes (Hypoxia-induced, Stress-induced, Senescence-associated, Cancer-associated, etc.) (Jarroux et al., 2017). Although the expression of lncRNAs are lower and more tissue-specific than mRNAs, they share certain similarities such as transcription by RNA Pol II, post-transcriptional modifications such as 5′ capping and 3′ polyadenylation (Bridges et al., 2021). LncRNAs have shown their ability to interact with several important cellular macromolecules. These interactions can often be attributed to the secondary structure of the RNA molecules and also directly correlate to its stability (Brown et al., 2012). The lncRNA interactome consists of proteins such as transcription factors acting as their guide, peptides, mRNAs, miRNAs, DNA, and important sites and molecules associated with the chromatin structure (Figure 1). Through these interactions, lncRNAs can control DNA replication, gene expression at an epigenetic, transcriptional and translational level and Protein stability, assembly and function (Khorkova et al., 2015).

## 2 Structure and function of lncRNA

The primary structure of lncRNAs is similar to that of protein-coding mRNAs, with both molecules going through similar processing and modifications (Bridges et al., 2021; Brown et al., 2012). However, the similarities end there, as lncRNAs lack the transcriptional flexibility of mRNAs, showing specific expression profiles and variable abundance in different cell lines, constraining researchers from studying their mode of action. LncRNAs also display poor evolutionary conservation, being riddled with unique base pair mismatches surrounding breaks of conserved sequences among species (Zampetaki et al., 2018). The tendency of lncRNAs to fold into secondary and higher-order structures that are thermodynamically stable is one of their key characteristics. RNA can create hydrogen bonds on the Watson-Crick face, the Hoogsteen face, and the ribose face. As a result of these collective interactions, RNA develops secondary structures such as double helices, hairpin loops, bulges, and pseudoknots, which are linked by higher-order tertiary interactions that are predominantly mediated by non-Watson-Crick base-pairing. As a result, coaxial stacks of helices that are arranged in parallel or orthogonal to one another dominate the structure of RNA. The sarcin-ricin loop, the K-turn, and the C-loop are recurring structural motifs present in the lncRNA molecules. Furthermore, these modular secondary structures typically fold initially and independently before subsequent tertiary interactions take place, leading to the hierarchical assembly of RNA structure (Mercer and Mattick, 2013). The secondary configuration of lncRNAs actually helps to establish its functional aspects. LncRNAs have a role in many different biological processes. These include - a) interacting with chromatin complexes and thereby contributing to the regulation of epigenetic genes, b) acting as modulators of proteins or multi-protein complexes, c) interacting with DNA/RNA-associated proteins to control transcriptional expression, d) regulating DNA stability through the formation of R-loop and triple helix, and e) assisting in the development of a higher-order chromatin structure (Graf and Kretz, 2020). Several methods of structure elucidation of lncRNAs are available nowadays, such as enzymatic probing methods like PARS (parallel analysis of RNA structure), fragmentation sequencing and chemical probing methods like DMS (Dimethyl Sulfate) Probing, SHAPE (selective 2′-hydroxyl acylation by primer extension), SHAPE-MaP (SHAPE and mutational profiling), icSHAPE-seq (*in vivo* click SHAPE sequencing) etc. that are being used to bridge the structure-function gap of lncRNA interactome (Zampetaki et al., 2018; Graf and Kretz, 2020).

## 3 Association of lncRNA in human pathobiology

The role of lncRNAs in the disruption of normal cellular operation of different organs and their involvement in the disease processes have been the subject of extensive exploration, and various mechanisms connecting the expression and interaction of lncRNAs to cellular dysfunction have been identified. The involvement of lncRNAs in disease pathobiology can be attributed to its interactions with the cellular signalling pathways governing inflammation, migration, apoptosis, autophagy and epigenetic modification.

### 3.1 Diabetes

In the hyperglycemic condition of diabetes, expression of lncRNA H19 is downregulated, and the overexpression of the non-coding RNA molecule has been shown to abrogate endothelial-mesenchymal transition related to diabetic retinopathy (Thomas et al., 2019) (Table 1). The opposite is true in the case of lncRNA HOTAIR, as the expression levels of the lncRNA in diabetic individuals were found to be significantly higher when compared to non-diabetic control. HOTAIR seemed to aggravate the angiogenic landscape of DR, increasing the expression of vascular endothelial growth factor (VEGF)-A, angiopoietin-like 4 (ANGPTL4), placental growth factor (PGF), hypoxia-inducible factor (HIF), interleukin-1 beta (IL-1β) and promote mitochondrial dysfunction and oxidative damage, all of which were alleviated by the knockdown of the RNA molecule (Biswas et al., 2021) (Table 1). Exosome-transmitted lncRNA LOC100132249 promoted endothelial dysfunction in diabetic retinopathy by sponging miR-199a-5p, which increased the activation of Wnt/β-catenin pathway, ultimately culminating in the regulation of endothelial-mesenchymal transition promoter SNAI1 (Hu Z. et al., 2023). Kumar and Datta in 2022 demonstrated that the upregulation of H19 in skeletal muscles in mice promoted insulin resistance in type 2 diabetes via increased IRS1 and downregulated HDAC6 expression. Furthermore, H19 inhibition normalized HDAC6 levels and protected the muscles from diabetes-mediated insulin dysfunction (Kumar and Datta, 2022). Increased levels of circulating lncRNAs NONHSAT054669.2 and ENST00000525337 were found in pregnant women suffering from gestational diabetes mellitus (GDM) and such lncRNAs can be used as an early biomarker for GDM (Jiang et al., 2023). Manna et al., in 2022 also showed that increased levels of lncRNA TUG1 combined with hsa-miR-607 and hsa_circ_0071106 in peripheral blood can be used as a prognostic marker type 2 diabetes mellitus, showing 75.2% combined sensitivity of diagnosis at and 100% specificity (Su et al., 2022). In high fat diet-induced GDM mice, TUG1 depletion promoted the expression of miR-328-3p, resulting in the activation of SREBP-2-mediated extracellular signal-regulated kinase (ERK) signaling pathway, causing insulin resistance and apoptosis of pancreatic islet cells. Upregulation of TUG1 promoted competitive binding with the target miR-328-3p, normalizing the expression of SREBP-2 and protected the mice from insulin resistance and apoptosis (Tang et al., 2023). Increased expression of LncRNA TUNAR (TCL1 upstream neural differentiation-associated RNA) in β-cells of patients with T2DM have shown positive correlation with the Wnt pathway as demonstrated by Zhou et al., in 2021. TUNAR suppressed Wnt antagonist Dickkopf-related protein 3 (DKK3) via interaction with histone modifier enhancer of zeste homolog 2 (EZH2), causing upregulation of Wnt pathway and regulate β-cell proliferation (Zhou et al., 2021) (Table 1).

**TABLE 1:** Roles of lncRNAs in the disruption of normal cellular operations and their involvement in various disease processes.

Disease category	lncRNA	Mechanisms and functions	References
Diabetes	H19	Downregulated in hyperglycaemia; overexpression abrogates endothelial-mesenchymal transition related to diabetic retinopathy	Thomas et al. (2019)
		Upregulated in Skeletal muscles; promote insulin resistance via increased IRS1 expression and decreased HDAC6 levels	Kumar and Datta (2022)
	HOTAIR	Upregulated in diabetes; aggravates angiogenesis in diabetic retinopathy, increases VEGF-A, ANGPTL4, PGF, HIF, IL-1β, and promotes mitochondrial dysfunction and oxidative damage	Biswas et al. (2021)
	LOC100132249	Promotes endothelial dysfunction in diabetic retinopathy via sponging of miR-199a-5p and activation of Wnt pathway	Hu et al. (2023a)
	NONHSAT054669.2 and ENST00000525337	Can act as a biomarker for gestational diabetes mellitus	Jiang et al. (2023)
	TUG1	Interacts with miR-328-3p and regulate SREBP-2 mediated ERK signalling pathway; can also act as a biomarker for T2DM.	Su et al. (2022), Tang et al. (2023)
	TUNAR	Regulates β-cell proliferation in T2DM via suppression of Wnt antagonist Dickkopf-related protein 3 (DKK3)	Zhou et al. (2021)
Cardiovascular Diseases	MALAT1	Associated with endothelial cells and cardiomyocytes in CVD; interacts with miRNAs (miR-145, miR-22-3p, miR-155, miR-503, miR-214, miR-92a); affects plaque formation, inflammation, hypertension, angiogenesis; stabilizes SREBP-1c protein; regulates atherosclerosis, myocardial infarction, inflammation, cardiac remodeling	Zeng et al. (2018), Zhao et al. (2016), Wang and Zhou (2018), Zhao et al. (2017), Song et al. (2018), Tang et al. (2015), Yan et al. (2020)
	MIAT	Overexpressed in high glucose conditions; regulates endothelial cells function via miR-150-5p/VEGF feedback loop; involved in cardiac fibrosis post-infarction	Yan et al. (2015), Qu et al. (2017)
	NEAT1	Enhances I/R injury by activating apoptosis and autophagy in diabetic mice’s cardiac cells	Ma et al. (2018)
	CARL	Inhibits mitochondrial fission by sponging miR-539; enhances PHB2 mRNA interaction, downregulates apoptosis	Wang et al. (2014)
	APF	Acts as a decoy for miR-188-3p; promotes binding with ATG7, regulating autophagic cell death in CAD-associated I/R injury	Wang et al. (2015)
	SENCR	Downregulated in high glucose; overexpression reverses inhibition of smooth muscle cell proliferation and migration	Zou et al. (2015), Wang and Sun (2020)
	TINCR	Associated with NLRP3-mediated pyroptosis in diabetic cardiomyopathy and regulated by METTL14 expression	Meng et al. (2022)
	ZFAS1	Acts as a ceRNA for miR-150-5p, regulating Cyclin D2 (CCND2) expression	Ni et al. (2021)
	GAS5	Upregulated CYP11B2 expression in cardiomyocytes; promote myocardial damage and apoptosis	Zhuo et al. (2021)
Kidney Diseases	MALAT1	Increased expression in type 2 diabetes-related kidney disease; aggravates renal fibrosis via miR-145/FAK axis; knockdown ameliorates hypoxia-induced kidney damage via miR-204/APOL1/NF-κβ signaling	Zhou et al. (2020), Liu et al. (2020), Lu et al. (2021)
	TUG1	Associated with diabetic kidney disease; protects against high glucose-induced damage by regulating TIMP3, sponging miR-377; alleviates LPS-induced podocyte damage via miR-197/MAPK pathway; ameliorates I/R injury by inhibiting apoptosis via miR-494-3p/E-cadherin activation	Li and Susztak (2016), Long et al. (2016), Wang et al. (2019a), Duan et al. (2017), Zhao et al. (2019a), Chen et al. (2021a)
	MEG3	Overexpression linked to hypoxia-induced kidney injury via miR-181b/TNF-α; silencing alleviates kidney injury	Pang et al. (2019)
	XIST	Targets and downregulates miR-217; downregulation alleviates podocyte apoptosis and kidney injury via miR-217/TLR4 axis; inhibits apoptosis and inflammation in renal fibrosis via miR-19b/SOX6 pathway	Jin et al. (2019), Xia et al. (2021), Huang et al. (2014), Ma et al. (2021)
	DLX6-AS1	Linked with albuminuria; promoted cellular damage and inflammatory responses in podocytes through miR-346-mediated regulation of GSK-3β pathway	Guo et al. (2023)
	ENST00000436340	Promoted diabetic kidney disease progression and podocyte damage by interacting with PTBP1	Hu et al. (2023b)
Inflammatory Diseases	ANRIL	Overexpressed in ulcerative colitis; aggravates inflammation via miR-323b-5p/TLR4/MyD88/NF-κβ pathway	Qiao et al. (2019)
	TUG1	Protective effect on intestinal tissue in ulcerative colitis by targeting miR-142-3p/SOCS1	Han et al. (2020)
	GAS5	Connected with glucocorticoid responses in pediatric IBD.	Lucafò et al. (2018)
	CCAT1	Promotes IBD-induced malignancy via MLCK activity and miR-185-3p	Ma et al. (2019)
	Various lncRNAs	Involved in atherosclerosis (e.g., SNHG12, MeXis, LeXis, MANTIS, NEXN-AS1, ANRIL, MEG3, CHROME, CERNA1: atheroprotective; GAS5, LASER, CCL-2, SMILR, TUG1, MAIT, NEAT1: atherogenic)	Arslan et al. (2017), Josefs and Boon (2020), Li et al. (2016)
	Various lncRNAs	Dysregulation linked to rheumatoid arthritis (e.g., HOTAIR, H19, LOC100652951, LOC100506036, LincRNA-p21, NR024118, C5T1, MALAT1, MEG3, NEAT1, ZFAS1, GAS5); associated with pro-inflammatory cytokines and MMPs	Miao et al. (2021), Li et al. (2018)

### 3.2 Cardiovascular diseases

The risk factors associated with cardiovascular diseases, such as high glucose/diabetic condition, lipid accumulation, cytokines and oxidative stress (Ross et al., 2019), can be linked to the regulatory expression of lncRNAs. Metastasis-associated lung adenocarcinoma transcript 1, also known as MALAT1, mainly associated with cancer, has shown significant expression in endothelial cells and cardiomyocytes in response to CVD risk factors (Zeng et al., 2018; Zhao et al., 2016; Wang and Zhou, 2018) and was intimately connected with the pathophysiological processes of CVD including autophagy, apoptosis and pyroptosis (Zhao et al., 2017; Song et al., 2018; Tang et al., 2015). MALAT1 interacted with various miRNAs such as miR-145, miR-22-3p, miR-155, miR-503, miR-214, and miR-92a associated with plaque formation, inflammation, hypertension, angiogenesis, activates Wnt/β-catenin signalling causing increased endothelial-mesenchymal transition, promote lipid accumulation by stabilising SREBP-1c protein, leading to regulation of atherosclerosis and also play a huge role in myocardial infarction, inflammation and cardiac remodelling (Yan et al., 2020) (Table 1). Myocardial infarction-associated transcript (MIAT) is another lncRNA involved in myocardial diseases. It has overexpression in patients with high glucose conditions and regulates the functions of endothelial cells via the miR-150-5p/VEGF feedback loop (Yan et al., 2015) (Table 1). MAIT also played a huge role in cardiac fibrosis after infarction, as its expression increased in mouse infarction models and its knockdown improved cardiac condition by inhibiting collagen production and proliferation of cardiac fibroblasts (Qu et al., 2017). NEAT1, or Nuclear-enriched abundant transcript 1, is a lncRNA linked to coronary artery disease (CAD)-associated ischemia/reperfusion (I/R) injury. NEAT1 enhanced I/R injury by activating both apoptosis and autophagy in the cardiac cells of diabetic mice (Ma et al., 2018) (Table 1). CARL (Cardiac apoptosis-related lncRNA) inhibited mitochondrial fission by sponging miR-539 and enhancing its interaction with prohibitin 2 (PHB2) mRNA, downregulating apoptosis (Wang et al., 2014) (Table 1) and APF (Autophagy-promoting factor-related lncRNA) act as a decoy for miR-188-3p, furthering its binding with ATG7, regulating autophagic cell death in CAD-associated I/R injury (Wang et al., 2015) (Table 1). SENCR or Smooth muscle and endothelial cell-enriched migration/differentiation-associated long noncoding RNA, associated with T2DM, have shown downregulation in db/db mice exposed to high glucose. Overexpression of SENCR seemed to play an important role in diabetic cardiomyopathy as it reverses the inhibition of mouse vascular smooth muscle cell proliferation and migration by high glucose levels (Zou et al., 2015; Wang and Sun, 2020) (Table 1). Meng et al. (2022) studied the effects of the expression of lncRNA TINCR (Terminal differentiation-induced non-coding RNA) and methyltransferase METTL14 in DCM-induced rat tissues. Downregulation of METTL14 enhanced TINCR expression in the cardiomyocytes and enhanced pyroptosis in diseased animals. METTL14 upregulation promoted m6A methylation of TINCR gene, inhibiting its expression and also reduced pyroptosis by suppressing NLRP3 expression (Meng et al., 2022). LncRNA ZFAS1 (ZNFX1 antisense RNA1) showed significant association with diabetic cardiomyopathy in db/db mice. ZFAS1 acts as a ceRNA for miR-150-5p, which promote Cyclin D2 (CCND2) expression and knockdown of ZFAS1 lead to reduced collagen deposition, decreased apoptosis and ferroptosis and alleviated DCM progression (Ni et al., 2021). Expression of lncRNA GAS5 (Growth arrest specific 5) showed increment along with the upregulation of CYP11B2 in high glucose-induced AC16 cardiomyocytes and a streptozotocin (STZ)-induced rat diabetes model as shown by Zhou et al., in 2021. Moreover, attenuation of GAS5 inhibited high glucose-induced myocardial damage and apoptosis by targeting miR-138 and downregulating CYP11B2 expression (Zhuo et al., 2021) (Table 1).

### 3.3 Kidney diseases

In the case of renal pathology, the Majority of kidney diseases stem from overlapping conditions that affect other parts of the body, such as diabetes and hypertension and can also be tissue-specific, such as acute injury, inflammation, glomerular, mesangial and tubulointerstitial diseases (Guo et al., 2019; Ignarski et al., 2019). MALAT1 showed increased expression in type 2 diabetes-related kidney disease, acting as a diagnostic marker and therapeutic target (Zhou et al., 2020). The non-coding RNA molecule has been shown to aggravate renal fibrosis by sponging miR-145 and interrupting the miR-145/FAK axis that regulates TGF-β activity (Liu et al., 2020). Furthermore, the knockdown of MALAT1 ameliorated the progression of hypoxia-induced acute kidney damage via upregulation of miR-204 and inhibition of APOL1/NF-κβ signalling (Lu et al., 2021) (Table 1). Taurine upregulated gene 1, or TUG1, is another lncRNA that shows an association with diabetic kidney diseases and can be correlated to disease-specific damage in the kidney due to sepsis and ischemia/reperfusion (I/R) stress. TUG1 expression decreased in diabetic podocytes, which caused detrimental metabolic changes in mitochondrial structure and function by regulating PGC-1α expression levels and led to energy depletion and increased ROS generation, ultimately culminating in diabetic kidney disease (Li and Susztak, 2016; Long et al., 2016). TUG1 expression protects kidney cells from high glucose-induced damage by promoting overexpression of TIMP3 through interaction with miR-21 (Wang F. et al., 2019), preventing accumulation of ECM proteins like fibronectin, TGF-β, PAI-1 and type-4 collagen by sponging miR-377 (Duan et al., 2017) in diabetic nephropathy, ameliorated LPS-induced podocyte damage by downregulating miR-197 and MAPK expression (Zhao D. et al., 2019) and alleviated I/R-mediated acute kidney injury by inhibiting apoptosis through interaction with miR-494-3p and activation of E-cadherin in HK2 cells (Chen L. et al., 2021) (Table 1). MEG3 or maternally expressed gene 3 overexpression in acute renal allografts demonstrated hypoxia-induced kidney injury by sponging miR-181b and upregulating TNF-α activity, all of which was alleviated by silencing MEG3 using siRNA (Pang et al., 2019) (Table 1). LncRNA X-inactive specific transcript or XIST have intimate ties to kidney diseases. XIST targets and downregulates mir-217, a negative regulator of TLR4 and downregulation of XIST alleviated podocyte apoptosis and kidney injury in membranous nephropathy by mir-217/TLR4 axis (Jin et al., 2019). Knockdown of XIST also inhibited apoptosis and inflammation caused by renal fibrosis in mice model by mir-19b mediated downregulation of SOX6 (Xia et al., 2021), and several studies demonstrated XIST as a potential biomarker for diseases related to glomerular nephropathy (Huang et al., 2014; Ma et al., 2021) (Table 1). Guo et al. (2023) investigated the role of a novel lncRNA DLX6-AS1 in diabetic nephropathy. They found that DLX6-AS1 expression in DN patients was linked with the extent of albuminuria and overexpression of the RNA molecule promoted cellular damage and inflammatory responses in podocytes through miR-346-mediated regulation of the Glycogen Synthase Kinase (GSK)-3β pathway whereas knockout of the lncRNA ameliorated glomerular podocyte injury and albuminuria (Guo et al., 2023). Another lncRNA, ENST00000436340 promoted diabetic kidney disease progression and podocyte damage by interacting with poly-pyrimidine tract binding protein 1 (PTBP1), promoting RAB3B mRNA degradation, and thereby causing cytoskeleton rearrangement and inhibition of GLUT4 translocation to cell membrane (Hu J. et al., 2023) (Table 1).

### 3.4 Inflammatory diseases

Owing to its property to interact with various cellular macromolecules and regulate the pathways related to immune response and inflammation, lncRNAs can be traced to have significant roles in inflammatory disease processes (Liao et al., 2018). In the context of inflammatory bowel diseases, lncRNA ANRIL, a 3.8K nucleotide-long antisense lncRNA present in the INK4 loci, demonstrated overexpression in ulcerative colitis and aggravated inflammation by negatively regulating miR-323b-5p, which itself is a negative regulator of TLR4/MyD88/NF-κβ pathway (Qiao et al., 2019) (Table 1). TUG1 showed a protective effect on intestinal tissue, both *in-vivo* and *in-vitro*, in ulcerative colitis by inhibiting inflammation-induced ROS and pro-inflammatory cytokines through targeting miR-142-3p and enhancing SOCS1 production (Han et al., 2020) (Table 1). lncRNA Growth Arrest Specific 5 or GAS5 display connection with glucocorticoid responses in pediatric IBD (Lucafò et al., 2018) (Table 1) and CCAT1 or Colon Cancer-associated Transcript 1 promotes IBD-induced malignancy by enhancing myosin light chain kinase (MLCK) activity and downregulating miR-185-3p (Ma et al., 2019) (Table 1). Atherosclerosis, associated with the buildup of fatty plaque on the arterial walls and localised monocyte activation and inflammation, has intricate linkage with the differential expression of lncRNAs in patients (Arslan et al., 2017). Several long non-coding RNA molecules such SNHG12, MeXis, LeXis, MANTIS, NEXN-AS1, ANRIL, MEG3, CHROME, CERNA1 show atheroprotective effects, whereas atherogenic lncRNA molecules like GAS5, LASER, CCL-2, SMILR, TUG1, MAIT, NEAT1 promote plaque buildup and inflammation via transcriptional and epigenetic regulation and protein modification in smooth muscle cells, endothelial cells and macrophages (Josefs and Boon, 2020; Li et al., 2016) (Table 1). Autoimmune and inflammatory consequences of rheumatoid arthritis such as cartilage erosion and destruction, synovial hyperplasia, inflammatory joint fluid and synovium, marginal bone erosion can be linked to the dysregulation of lncRNAs like HOTAIR, H19, LOC100652951, LOC100506036, LincRNA-p21, NR024118, C5T1, MALAT1, MEG3, NEAT1, ZFAS1, GAS5. Such malregulations are associated with the overexpression of pro-inflammatory cytokines like IL-1β, IL-6, IL-8, IL-10 and matrix metalloproteinases like MMP2, MMP13, resulting in increased inflammation, collagen destruction, apoptosis and aggravation of arthritic condition (Miao et al., 2021; Li et al., 2018) (Table 1).

## 4 LncRNAs in cancer

Due to the diverse interactome of lncRNAs in the cellular and subcellular levels, it is shown to be involved in various important regulatory and developmental functions. But on the other hand, this also implicates lncRNAs as the driver of several disease processes. Many lncRNAs have undergone differential expression in various cancer types, establishing their signature in disease-associated development, dysregulation and damage. LncRNAs may control cell migration, invasion, apoptosis, proliferation, and stemness in cancer development. The abnormal development of lncRNAs and their participation in various cellular functions make them potential cancer therapeutic targets.

Cancer is a multistage disease comprising complex interactions with various cellular, subcellular and extracellular factors, ultimately culminating in the unregulated growth, immortalisation of cells, loss of adhesive capabilities, increase in cellular plasticity, destruction of tissue structure, inflammation, immune escape, spreading of dysregulated cells and tumour development (Hanahan, 2022). The hallmarks of cancer development can be traced back to four main events –

1) Insensitivity to growth-inhibiting factors and Self-sufficiency in growth-promoting factors
2) Epithelial-Mesenchymal Transition
3) Angiogenesis
4) Metastasis

### 4.1 LncRNAs regulating insensitivity to growth-inhibiting factors and self-sufficiency in growth-promoting factors

Regulation of growth factors is an important matric separating normal cellular growth from the cancerous phenotype. Normal cellular homeostasis is maintained by a highly controlled and steady balance of growth-promoting factors like epidermal growth factors (EGF), insulin-like growth factors (IGF), fibroblast growth factors (FGF) and vascular endothelial growth factors (VEGF) with tumor suppressors like TP53, PTEN, p21, p53, RB, etc. in the presence of external stimuli. Cancer cells differ from normal cells due to insensitivity to external stimulation, resulting in loss of cellular homeostasis, promoting self-sustaining growth signals and altered expression of growth inhibitors.

LncRNAs have shown remarkable crosstalk with the cellular growth-regulating proteome (Table 2). Decreased expression of lncRNA H19 promoted endometriosis in women via downregulation of IGF signalling via H19/Let-7/IGF1R axis. H19 acted as a ceRNA for miRNA Let-7, which, in turn, targeted IGF1R. Overexpression of H19 decreased Let-7 expression and upregulated the mRNA levels of IGF1R, promoting endometrial cell growth (Ghazal et al., 2015). Vennin et al., in 2015 also demonstrated that increased H19 levels promoted the tumorigenesis of breast cancer via upregulation of its by-product, miRNA-675, which led to the decreased expression of ubiquitin ligase E3 family proteins - c-Cbl and Cbl-b (Vennin et al., 2015). Furthermore, H19-derived miRNA-675 has been reported to inhibit the expression of tumour suppressor RB in colorectal cancer, promoting tumour growth (Tsang et al., 2010) and displaying cancer cell-proliferating activity in bladder cancer by inhibiting p53 activation and decreasing Bax/Bcl-2 ratio (Liu et al., 2016). The involvement of lncRNA CAR intergenic 10 (CAR10) in the growth of lung cancer was studied by Wei et al. (2016). The study showed that the binding of CAR10 with Y-box-binding protein 1 (YB-1) stabilizes the transcription factor, resulting in the increased expression of EGFR, enhancing cancer growth. Downregulation of the RNA molecule in A549 cells *in vivo* suppressed cell growth and inhibited the expression of YB-1 and EGFR (Wei et al., 2016). Lower levels of lincRNA-p21 found in liver cirrhosis and fibrosis directly correlate to the decreased expression of tumor suppressor p21. Upregulation of lincRNA-p21 enhanced p21 expression and inhibited cell-cycle progression and proliferation of primary hepatic stellate cells (HSCs) (Zheng et al., 2015). Sun et al. (2016) associated the increased expression of lncRNA FGFR3-AS1 with tumor growth, metastasis, and poor survival in osteosarcoma. The RNA molecule targeted the 3′ UTR of its antisense transcript, FGFR3, promoting its expression and downregulation of FGFR3-AS1 inhibited tumor proliferation *in-vitro*. LncRNA PTENP1 showed tumour-suppressive effects in gastric cancer by sponging miR-106b and miR-93, upregulating the expression of tumour inhibitor PTEN (Zhang et al., 2017).

**TABLE 2:** LncRNAs regulating Insensitivity to growth-inhibiting factors and Self-sufficiency in growth-promoting factors.

Sl no.	Name of lncRNA	Type of disease/Cancer	Function	References
1	H19	Endometriosis	Sponges miRNA Let-7, promoting upregulation of IGF1R	Ghazal et al. (2015)
		Breast cancer	lncRNA-derived miRNA-675 targets ubiquitin ligase E3 family proteins - c-Cbl and Cbl-b, promoting tumor growth	Vennin et al. (2015)
		Colorectal cancer	miRNA-675 suppresses the expression of RB	Tsang et al. (2010)
		Bladder cancer	miRNA-675 Inhibits p53 expression and decrease Bax/Bcl-2 ratio	Liu et al. (2016)
2	CAR10	Lung cancer	Stabilizes YB-1, prompting increased transcription of EGFR	Wei et al. (2016)
3	LincRNA-p21	Liver cirrhosis and Fibrosis	Regulates expression of tumor suppressor p21	Zheng et al. (2015)
4	FGFR3-AS1	Osteosarcoma	Promotes increased expression of its antisense transcript, FGFR3	Sun et al. (2016)
5	PTENP1	Gastric cancer	Enhances the expression of tumor inhibitor PTEN by sponging miRNA-106b and miRNA-93	Zhang et al. (2017)

### 4.2 LncRNAs in epithelial-mesenchymal transition

Epithelial-mesenchymal transition is an important physiological process that mainly entails the change occurring in the adherent nature of epithelial cells and their transition into the mesenchyme. Although EMT is involved in several normal body functions such as embryogenesis, organ development, wound healing, etc., its deregulation can be implicated in diseases such as fibrosis and cancer. EMT is a marker of cancer growth and progression and can be associated with immunoresistant and chemoresistant attributes of some cancer types. The transition basically progresses with the loss of the epithelial polarity (Baso-apical polarity) of the abnormal cells, which results in the loss of their adhesion junctions, cascading to the degradation of the extracellular matrix. The transitioning cells also display downregulated epithelial surface markers such as E-cadherin, occludin, claudin-1 and upregulation of mesenchymal markers like α-SMA (Smooth Muscle Actin), fibronectin, vimentin (Fedele et al., 2022; Liu L. et al., 2022).

**FIGURE 2:**
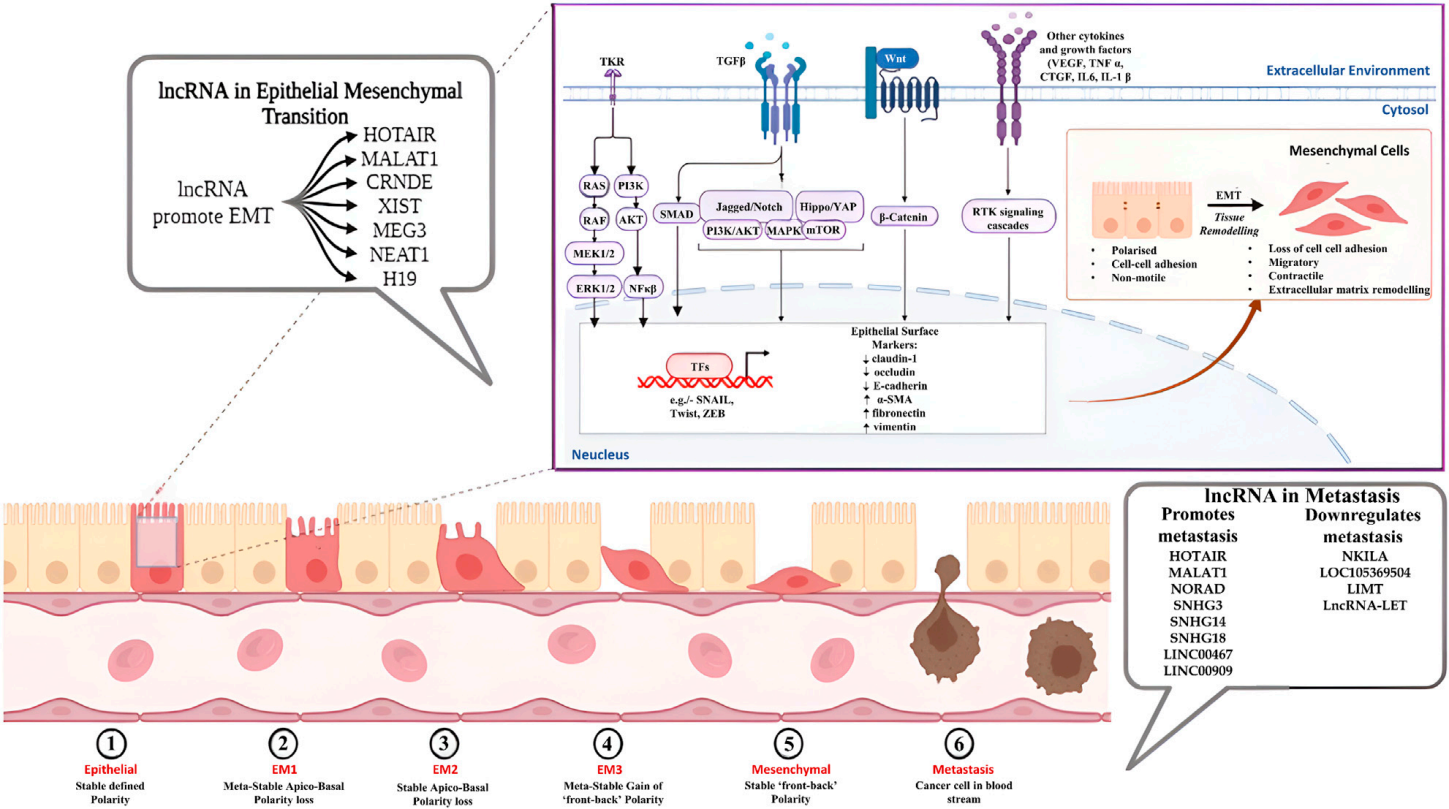
Role of lncRNAs in EMT and Metastasis.

Several cellular signalling pathways are involved in the regulation of the EMT process, including but not limited to transforming growth factor (TGF)-β/Smad, Wnt/β-catenin, PI3K/AKT, ERK/MAPK, p38 MAPK and JAK/STAT pathways, further leading to the regulation of EMT-specific transcription factors like SNAIL, TWIST and ZEB (Fedele et al., 2022; Fedorova et al., 2022). The involvement of non-coding RNA molecules like lncRNAs in EMT can be traced back to their crosstalk with the different cellular pathways involved in the dysregulation of the epithelial/mesenchymal marker expression, through interaction with other noncoding RNAs and also through the epigenetic changes brought about by the RNA molecules (Table 3; Figure 2). LncRNA HOTAIR (HOX Transcript Antisense RNA) have shown significant association with the progression of cancer through the process of EMT. Jarroux et al., 2021 investigated the role of HOTAIR in the epigenetic stimulation of EMT and found the interaction between the non-coding RNA and LSD1 (Lysine demethylase) significantly promoted epithelial cell migration via repressive methylation (H3K27) in the chromatin which was alleviated by the overexpression of LSD1 (Jarroux et al., 2021). Furthermore, HOTAIR promoted oxaliplatin resistance and EMT in colorectal cancer via negative regulation of miR-1277-5p and knockdown of HOTAIR significantly upregulated miR-1277-5p expression, increasing chemosensivity through inhibition of ZEB1 expression (Weng et al., 2022). Silencing of HOTAIR also decreased EMT in Pancreatic cancer cells by downregulating mesenchymal markers, increasing E-cadherin expression and inhibiting the Wnt/β-catenin pathway (Tang et al., 2021). In 2022, Wu et al. demonstrated that suppression of lncRNA MALAT1 (Metastasis-Associated Lung Adenocarcinoma Transcript 1) inhibited the cancer stem cell (CSC)-like properties of laryngocarcinoma cells via miR-708-5p/YAP1/BRD4 axis (Wu et al., 2022). The epigenetic link of MALAT1 to EMT was uncovered by Zhao et al., showing N6-methyladenosine (m6A)-methyltransferase METTL3 upregulated EMT in breast cancer by overexpression of MALAT1. MALAT1 act as a sponge for miR-26b, which further interacts with HMGA2 (High Mobility Group A2) to promote EMT. Silencing METTL3 suppressed EMT by inhibiting MALAT1 expression, establishing the MALAT1/METTL3 axis of EMT in breast cancer (Zhao et al., 2021). Wang et al., 2022 demonstrated the potential of lncRNA CRNDE (Colorectal neoplasia differentially expressed) as a viable target for inhibiting the spread of ovarian cancer. CRNDE has shown differential expression along with its counterpart miR-423-5p in ovarian cancer cell lines and played a role in the expression of a major oncoprotein, FSCN1. Moreover, silencing of the CRNDE/FSCN1 axis stopped the extracellular matrix degradation and EMT in cancer cells via inhibition of MMP-2 and MMP-9 (Wang Q. et al., 2022). LncRNA XIST (X-inactive specific transcript) showed association with osteosarcoma EMT by acting as a bridge between Human antigen R (HuR) and argonaute RISC catalytic component AGO2. Silencing of HuR suppressed AGO2 expression, and decreased AGO2 inhibited the EMT and migration of OS cells (Liu Y. et al., 2021). In 2022, Gu et al. combined an *in silico* approach with *in-vitro* studies to determine the connection of lncRNA MEG3 (Maternally expressed gene 3) with the migration and EMT of breast cancer. The study showed reduced MEG3 expression in a Pan-cancer context and also demonstrated the reduced expression profile of the non-coding RNA molecule in high-invasive breast cancer cells compared to low-invasive cells. Furthermore, the expression of MEG3 was tightly correlated with the expression of Schlafen-5 (SLFN5), and overexpression of MEG3 ameliorated EMT in highly invasive breast cancer cells by modulating SLFN5 expression via MEG3/miR-146b-5p/SLFN5 axis (Gu et al., 2022). Downregulation of MEG3 also promoted sequestosome 1 (SQSTM1) expression in nasopharyngeal carcinoma cells and is responsible for the increased migration, invasion and EMT in NPC cells (Zhou C. et al., 2022). The promotional effects of NEAT1 (Nuclear paraspeckle assembly transcript 1) in the invasiveness and migration of osteosarcoma cells were shown by Chen et al. (2021a). NEAT1 enhanced EMT in OS cells by sponging miR-483, which targets STAT3 in the 3′ UTR region. The binding of miR-483 with NEAT1 increased STAT1, STAT3 and other EMT markers in U2O3 cells and greatly helped in EMT and metastasis of cancer cells (Chen et al., 2021b). Additionally, silencing of NEAT1 also inhibited EMT in retinoblastoma cells by upregulating miR-24-3p expression, which further targets LRG1 (leucine-rich-α-2-glycoproteins) and downregulating EMT markers such as N-cadherin, vimentin (Luan et al., 2021). LncRNA H19 has been shown to regulate ovarian cancer migration via interaction with non-coding RNA miR-140-5p. The lncRNA exhibited sponging of miR-140-5p, activating the overexpression of the PI3K/AKT pathway and promoting EMT and migration (Xu H. et al., 2021). H19 also participated in the promotion of EMT and aggressiveness of gastric cancer by inducing the translocation of β-catenin into the nucleus, thereby activating the Wnt/β-catenin pathway (Liu J. et al., 2022). LncRNA LITATS1 among others is a critical player of EMT in breast and non-small cell lung cancer cells which is also a keeper of epithelial integrity as well as suppressor of TGF-β/SMAD signaling. This suppression further results in cytoplasmic retention of SMURF2 and subsequently inhibiting EMT and cell migration. This role of lncRNA LITATS1 potentiates its effectiveness for a favorable survival outcome for breast and small cell lung cancer patients (Fan et al., 2023a). Contrastingly, lncRNA LETS1 is an activator of TGF-β induced EMT contributing to the oncogenicity of breast and lung cancer by increasing cancer cell migration and extravasation. LETS1 was found to interact with a transcription factor NEAT5, which in turn protected the TβRI receptor from degradation creating a positive feedback loop for TGF-β signaling and initiating EMT downstream (Fan et al., 2023b). LncRNA MIR200CHG is another candidate that sponges a microRNA miR-200c, protecting it from degradation and inhibiting EMT downstream while other findings in this regard suggest that MIR200CHG modulates miR-429 further regulating EMT in gastric cancer. Reports regarding lncRNA MIR200CHG has been contradictory considering its role in regulating EMT in other cancer types but in case of Microsatellite stable/epithelial-mesenchymal transition subtype of gastric cancer it has been established with solid evidence that MIR200CHG is a master regulator of EMT and promoter hypermethylation-mediated MIR200CHG silencing is attributed to poor prognosis in gastric cancer patients (Zhu et al., 2023).

**TABLE 3:** LncRNAs in epithelial-mesenchymal transition.

Sl no.	Name of lncRNA	Type of cancer	Function	References
1	HOTAIR	Colorectal cancer	Promotes oxaliplatin resistance via miR-1277-5p/ZEB1 axis	Weng et al. (2022)
		Pancreatic cancer	Upregulates Wnt/β-catenin pathway and promotes expression of mesenchymal markers	Tang et al. (2021)
2	MALAT1	Laryngocarcinoma Breast cancer	Targets miR-708-5p, modulating the expression of BRD4 (Bromodomain-containing protein 4) and YAP1 (Yes-associated protein 1)	Wu et al. (2022)
			METTL3 upregulated MALAT1 expression. MALAT1 sponge miR-26b, causing interaction with HMGA2	Zhao et al. (2021)
3	CRNDE	Ovarian cancer	Competitively binds to miR-423-5p and promote expression of Fascin actin-bundling protein 1 (FSCN1)	Wang et al. (2022a)
4	XIST	Osteosarcoma	Acts as a bridge between HuR and AGO2, supporting EMT	Liu et al. (2021a)
5	MEG3	Breast cancer	Inhibits EMT via modulating SLFN5 expression	Gu et al. (2022)
		Nasopharyngeal carcinoma	Downregulates SQSTM1 expression, ameliorating EMT	Zhou et al. (2022a)
6	NEAT1	Osteosarcoma	Binds to miR-483, enhancing expression of EMT markers	Chen et al. (2021b)
		Retinoblastoma	Repress miR-24-3p expression and promote overexpression of N-cadherin, vimentin	Luan et al. (2021)
7	H19	Ovarian cancer	Acts as ceRNA for miR-140-5p, activating PI3K/AKT pathway	Xu et al. (2021a)
		Gastric cancer	Upregulates Wnt/β-catenin pathway by promoting nuclear translocation of β-catenin	Liu et al. (2022b)
8	LITATS1	Breast and Small cell lung Cancer	Suppresses TGF-β/SMAD signaling, causing retention of SMURF2 and inhibiting EMT	Fan et al. (2023a)
9	LETS1	Breast and Lung Cancer	Promotes EMT by interacting NEAT5, which protects the TβRI receptor from degradation creating a positive feedback loop for TGF-β signaling	Fan et al. (2023b)
10	MIR200CHG	Gastric Cancer	Sponges miR-200c and miR-429, inhibiting EMT	Zhu et al. (2023)

### 4.3 LncRNAs in angiogenesis

Angiogenesis is the process of new blood vessel formation from pre-existing vasculature. It is an important physiological process that is regulated by the differential expression of Pro and Anti-angiogenic factors in the cellular microenvironment of growing organisms. Normally, migration and growth of mesoderm-derived endothelial cells bring about the angiogenic changes that mainly occur during embryonic development, wound healing, platelet formation, and menstrual cycle. However, physiological angiogenesis differs broadly from cancer angiogenesis as in physiological conditions, angiogenesis is tightly regulated by the balance of Pro and Anti-angiogenic factors as well as interactions with other cell types present in the vasculature such as pericytes, macrophages, endothelial cells and immune cells. Hypoxia-induced due to excessive tumour metabolism and growth produce optimal conditions for the production of pro-angiogenic factors like Vascular Endothelial Growth Factors (VEGFs), Platelet Derived Growth Factors (PDGFs), Angiopoietins, Matrix Metalloproteinases (MMP-2, 9), Interleukins (IL-1, 6, 10), Integrins and overexpression of such proteins activate the angiogenic switch in Tumor Endothelial Cells (TECs). In addition to the increased oxygen demand aggravating the angiogenic condition in tumours, Non-endothelial tumour cells can also participate in angiogenesis through the process of “vasculogenic mimicry,” where non-endothelial cells behave as endothelial cells due to the constant pro-angiogenic signals present in the microenvironment and form abnormal blood vessels. Angiogenesis in cancer directly correlates to tumour growth and is shown to promote metastasis (Ayoub et al., 2022; Ozel et al., 2022).

**FIGURE 3:**
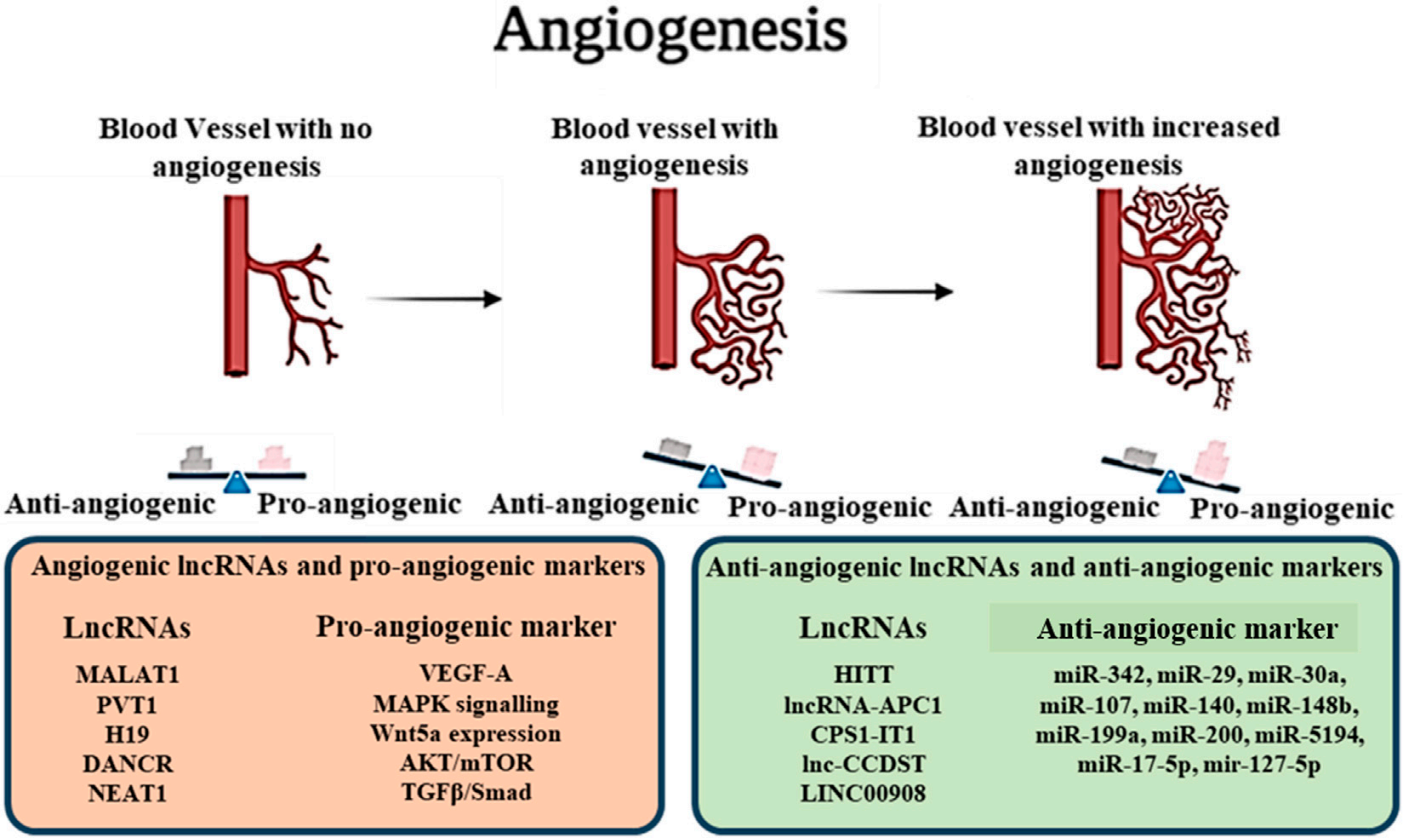
Role of lncRNAs in Angiogenesis.

The association of lncRNAs with angiogenesis can be traced back to the crosstalk of the non-coding RNA molecules with cellular pathways associated with oncogenesis, including PI3K/AKT/mTOR, STAT3, NF-κβ, ERK/MEK, all of which are significantly bound to the expression of proangiogenic VEGFs and also associated with the vasculogenic mimicry and immunosuppressive characteristics of cancer cells (Zhao J. et al., 2019) (Table 4; Figure 3). LncRNA MALAT1 showed significant association with the angiogenesis of osteosarcoma by targeting miR-150-5p, resulting in upregulation of VEGF-A, consequently increasing the production of critical pro-angiogenic factors (Vimalraj et al., 2021). MALAT1 also promoted angiogenesis in Multiple myeloma by modulating the miR-15a/16 expression and promoting tumorigenesis via the miR-15a/16/VEGFA axis (Yan et al., 2023). In malignant glioma cancer, a correlation between the expression of lncRNA PVT1 and angiogenesis has been observed. PVT1 targeted the expression of miR-1207-3P, which, in turn, regulated the production of Hepatocyte Nuclear Factor 1-β (HNF1-B), promoting angiogenesis and spreading of cancer (Bi et al., 2021). Zhou et al., 2022 studied the connection between the non-coding RNA transcriptome and the pathway promoting cellular migration and angiogenesis in glioma cells. The study observed that lncRNA H19 upregulated the expression of Wnt5a, a promoter of cellular migration and mediated angiogenesis by the overexpression of the Wnt/β-catenin axis. H19 also suppressed the expression of anti-angiogenic microRNA miR-342, which targeted the cellular migration pathways and further aggravated tumorigenesis (Zhou Q. et al., 2022). Furthermore, H19 has been observed to downregulate the expression of several anti-angiogenic microRNAs in smokers, including miR-29, miR-30a, miR-107, miR-140, miR-148b, miR-199a and miR-200. Such noncoding RNA molecules regulate the production of critical angiogenic factors like VEGFs, PDGFs and HIFs, leading to increased cellular migration and angiogenesis in several cancer types like bladder cancer, breast cancer, colorectal cancer, glioma, gastric adenocarcinoma, hepatocellular carcinoma, meningioma, non-small-cell lung carcinoma and oral squamous cell carcinoma (Shirvaliloo, 2023). Jia et al. demonstrated the involvement of lncRNA DANCR in the overexpression of pro-angiogenic signals in melanoma. The inherent property of lncRNAs as sponges for microRNAs came to play a huge role as DANCR targets miR-5194, an anti-angiogenic RNA molecule that regulates angiogenesis in normal conditions. Downregulation of miR-5194 promoted angiogenesis and tumour proliferation via overexpression of VEGF-B, which was suppressed by the knockdown of the lncRNA, validating the role of the non-coding RNA in cancer development (Jia et al., 2023). Increased transcription levels of lncRNA NEAT1 can be associated with the proliferation, aggressiveness and angiogenesis of several cancer types such as gastric cancer, ovarian cancer and hepatoma carcinoma due to the crosstalk with the autophagic AKT/mTOR pathway, its ability to act as a competing endogenous RNA (ceRNA) to anti-angiogenic miRNA molecules like miR-17-5p, mir-127-5p and the subsequent enhancement of pro-angiogenic signalling such as TGF-β/Smad, VEGFs, FGFs and MMPs (Xu Y. et al., 2021; Yuan et al., 2021; Guo J. et al., 2022). LncRNA BLACAT3 was found to have regulatory effects on the expression of NCF2/p67 phox which is clinically correlated with poor prognosis in bladder cancer patients. The angiogenic role of lncRNA BLACAT3 was further confirmed when BLACAT3 knockdown bladder cancer cells were unable to colonize and cause tumor formation, when xenografted in Balb/C nude mice (Xie et al., 2023). In a very recent study it was found that a novel lncRNA LOC101928222 was significantly upregulated in colorectal cancer (CRC) patients as well as in CRC cell lines. Further, it was found that LOC101928222 increased cholesterol synthesis significantly by modulating METTL16-mediated m6A-dependent pathways which lead to pro-angiogenic effects (Chang et al., 2024). Recently, exosomal lncRNAs gained attention in regards to their significant role in the oncogenic environment. Such as the lncRNA PART1 of EC9706 exosomes, which was found to be involved in oesophageal cancer angiogenesis by acting as a sponge of miR-302a-3p, setting CDC25A free which further resulted in cell cycle progression and tumor angiogenesis as evident by human umbilical vein endothelial cell proliferation *in vitro* (Ding et al., 2023). LncRNAs HITT and lncRNA-APC1 showed anti-angiogenic property in colorectal cancer (Wang et al., 2020; Wang et al., 2021). HITT was found to suppress angiogenesis by repressing HIF-1α expression through its YB-1-binding motif titrates which titrates away YB-1 from the 5′UTR of HIF-1α mRNA (Wang et al., 2020). Whereas lncRNA-APC1 interacts with Rab5b mRNA and decreases its stability which leads to inhibition of exosome production (Wang et al., 2021). CPS1-IT1 also showed antiangiogenic property in melanoma by inhibiting the expression of Cyr61 and eventually its downstream pro-angiogenic factors VEGF and MMP9 (Zhou et al., 2019). LncRNA-CCDST exhibited antiangiogenic property in cervical cancer by degradation of pro-oncogenic factor DHX9 through ubiquitin proteasome pathway (Ding et al., 2019). LINC00908 an antiangiogenic LncRNA found in TNBC, which encodes a polypeptide ASRPS, which reduces STAT3 phosphorylation and eventually inhibits VEGF expression (Wang Y. et al., 2019).

**TABLE 4:** LncRNAs in angiogenesis.

Sl no.	Name of lncRNA	Type of cancer	Function	References
1	MALAT1	Osteosarcoma	Targets miR-150-5p, resulting in the upregulation of VEGF-A	Vimalraj et al. (2021)
		Multiple myeloma	Regulates the expression of miR-15a/16 as ceRNA, promoting overexpression of VEGF-A	Yan et al. (2023)
2	PVT1	Glioma	Sponges miR-1207-3p, increasing HNF1-B expression, thereby enhancing MAPK signalling	Bi et al. (2021)
3	H19	Glioma	Suppresses miR-342 and promote Wnt5a expression	Zhou et al. (2022b)
4	DANCR	Melanoma	Promotes angiogenesis via inhibition of miR-5194 and encouraging expression of VEGF-B	Jia et al. (2023)
5	NEAT1	Gastric cancer, ovarian cancer and hepatocellular carcinoma	Interacts with different microRNAs such as miR-17-5p, miR-127-5p, modulating pathways like AKT/mTOR, TGF-β/Smad, resulting in promotion of angiogenesis	Xu et al. (2021b), Yuan et al. (2021), Guo et al. (2022a)
6	BLACAT3	Bladder cancer	Regulates expression of NCF2/p67 phox, promoting tumor formation	Xie et al. (2023)
7	LOC101928222	Colorectal cancer	Modulate METTL16-mediated m6A-dependent pathways which lead to pro-angiogenic effects	Chang et al. (2024)
8	PART1	Oesophageal cancer	Acts as a sponge of miR-302a-3p, setting CDC25A free, resulting in cell cycle progression and tumor angiogenesis	Ding et al. (2023)
9	HITT	Colorectal cancer	Repress HIF-1α expression through its YB-1-binding motif and titrates away YB-1 from the 5′UTR of HIF-1α mRNA	Wang et al. (2020)
10	lncRNA-APC1	Colorectal cancer	interacts with Rab5b mRNA and decreases its stability which leads to inhibition of exosome production	Wang et al. (2021)
11	CPS1-IT1	Melanoma	Inhibits the expression of Cyr6 that eventually downregulated its downstream pro-angiogenic factors VEGF and MMP9	Zhou et al. (2019)
12	LncRNA-CCDST	Cervical cancer	Degrades pro-oncogenic factor DHX9 through ubiquitin proteasome pathway	Ding et al. (2019)
13	LINC00908	Triple Negative Breast Cancer	Encodes a polypeptide ASRPS, which reduces STAT3 phosphorylation and eventually inhibits VEGF expression	Wang et al. (2019b)

### 4.4 LncRNAs in metastasis

Metastasis is the process of progression of cancer where cancer cell cells from the primary tumour site spread throughout the body and colonise other parts to form secondary tumours. It is the deadliest event to occur during cancer development and is the collective consequence of the complex cross-reactions between the tumour cells and the tumour microenvironment. Metastasis is a multistage event initiated from the start of tumorigenesis. After tumour formation, cancer cells degrade the extracellular matrix using proteases such as MMPs, exposing the cancer cells to the mesenchyme and promoting EMT of the cancer cells. Increased oxygen requirement of the tumour due to its enhanced growth and metabolism creates a suitable hypoxic condition, and interactions with the endothelial cells in such conditions create the environment for angiogenesis. The transitioned cancer cells then invade the newly formed vessels via intravasation, allowing the cancer to access the bloodstream, from where it spreads across the body, protecting itself from the host immune responses by modulation of self-antigens, secreting chemokines to modulate immune cell responses and recruit immuno-suppressive cells to counteract the immune system. When the site of the secondary infection is reached, the cancer cells invade the tissue surrounding the vessels by extravasation and begin the cycle of new tumour formation, leading to the relapse of the disease. EMT, Angiogenesis, impaired autophagic and apoptotic pathways, immune escape and suppression–all the events leading to cancer growth and progression all culminate in metastasis (Majidpoor and Mortezaee, 2021; Neophytou et al., 2021; Benboubker et al., 2022).

LncRNAs participate in the metastasis of cancer in both active and passive manner. Passively, lncRNA molecules have been shown to affect key processes associated with the metastatic machinery, such as the upregulation of EMT and angiogenic pathways by modulating epigenetic and transcriptional changes, encouraging migration and invasion and regulating autophagic and inflammatory pathways, increasing cancer cell survivability. On the other hand, the non-coding RNAs also play an active role in promoting a metastatic microenvironment by modulating immune responses in immune cells via the production of cytokines and chemokines, enhancing immune escape and viability of tumour cells and stimulating metastatic colonisation of migratory cells into the secondary tumour sites in the body (Liu SJ. et al., 2021) (Table 5; Figure 2). LncRNA HOTAIR has been shown to regulate autophagic and cellular growth responses in breast cancer by competitively binding with the non-coding miR-130a-3p. MiR-130a-3p modulated the metastatic spreading of the carcinoma via Suv39H1/AKT/mTOR axis and sponging of the non-coding RNA by HOTAIR upregulated Suv39H1 responses, promoting cancer cell growth and metastasis (He W. et al., 2022). Li et al. further demonstrated the effects of HOTAIR on cancer metastasis by regulation of hepatocellular adhesion molecule (hepaCAM). Expression of hepaCAM was inversely related to the HOTAIR in prostate cancer due to repression of hepaCAM promotor by HOTAIR-mediated recruitment of PRC2 and downregulation of hepaCAM promoted metastasis by the abnormal activation of MAPK signalling pathway. HOTAIR depletion regenerated hepaCAM levels in the cells and restricted cancer growth and metastasis, indicating HOTAIR/hepaCAM/MAPK axis to be a potential therapeutic roadway for prostate cancer (Li et al., 2021a). Knockdown of MALAT1 suppressed proliferation and metastasis of head and neck squamous cell carcinoma by upregulating Von Hippel–Lindau tumour suppressor (VHL). Downregulation of MALAT1 decreased the expression of cell migratory factors like n-cadherin, vimentin and SNAIL, stabilised the β-catenin and NF-κβ pathways by regulating the expression of β-catenin and p65 and promoted apoptosis in tumour cells by upregulating cleaved caspase three and PARP, such effects being reversed by the downregulation of VHL (Duan et al., 2023). The expression of NF-κB-interacting lncRNA (NKILA) depreciated in several cancer types, such as oral squamous cell carcinoma and oesophageal squamous cell carcinoma. Overexpressing NKILA attenuated cancer metastasis and tumorigenesis by blocking the translocation of NF-κB component p65 from the cytoplasm into the nucleus and inhibiting the phosphorylation of IκBα, thereby downregulating NF-κB signalling. Furthermore, NKILA also regulates TGFβ signalling and has been shown to decrease MMP14 levels in cancer, resisting cancer migration and metastasis (Hu et al., 2020; Lu et al., 2018). In a recent study, a novel lncRNA LOC105369504 was identified, which was found to be a potential functional lncRNA having tumor suppressive as well as antimetastatic activity in colorectal cancer cells by regulating the protein of paraspeckles compound 1 (PSPC1) (Zhan et al., 2023). Another lncRNA LIMT (lncRNA inhibiting metstasis) also known as LINC01089 also found to inhibit the proliferation and metastasis of different cancers like colorectal cancer, breast cancer, gastric cancer and hepatocellular cancer (Li and Guo, 2020; Yuan et al., 2019; Guo and Li, 2020; Hu et al., 2022). LncRNA-LET showed antimetastatic activity through its association with NF90, which is known to promotes tumorigenicity through PI3K/Akt pathway (Yang et al., 2013). LncRNA NORAD showed conflicting associations in tumour growth, cancer progression and metastasis. In breast cancer, cellular levels of NORAD were downregulated compared to the control. Overexpression of the non-coding RNA further showed antimetastatic and anti-proliferative effects on breast cancer cells by increasing DNA damage and apoptosis via sponging miR-155-5p. The increment of miR-155-5p levels was observed in breast cancer cell lines, and targeting of mir-155-5p by NORAD positively regulated the tumour suppressor protein SOCS1, showing ameliorative effects *in vitro* (Liu W. et al., 2021). Inversely, Mao et al. investigated the role of NORAD in Lung cancer progression and found that lncRNA was strongly expressed in the lung cancer cell lines compared to normal cells. Mechanistically, they showed NORAD to be acting as a ceRNA to miR-28-3p. MiR-28-3p directly targets transcription factor E2F2, a well-known oncogene associated with poor survival in several cancer types such as breast cancer, ovarian cancer, retinoblastoma, and NORAD-mediated downregulation of the microRNA promote overexpression of the oncogenic transcription factor, leading to increased tumour growth and metastasis (Mao et al., 2022). The lncRNAs of the Small Nucleolar Host Gene (SNHG) family are found to have significant interactions with the pathways related to tumour proliferation, growth, invasion and metastasis. Xie et al., 2021 observed the role of lncRNA SNHG3 in the metastasis and growth of gastric cancer. SNHG3 targeted miR-139-5p, competitively binding to the microRNA molecule and preventing its association with transcription factor MYB, known to regulate cell growth, apoptosis and DNA damage repair. Sequestration of miR-139-5p upregulated MYB expression, promoting cancer growth, tumorigenesis and metastasis (Xie et al., 2021). Sponging of miR-206 by lncRNA SNHG14 promoted cancer proliferation and metastasis in hepatocellular carcinoma by overexpression of SOX9 as demonstrated by Lin et al. citing a strong connection of the non-coding RNA molecule with the tumorigenesis. They further annotated that the knockout of SNHG14 and SOX9 suppressed tumour migration and invasion by upregulation of miR-206 and increased apoptosis in cancer cells, all of which was reversed by the repression of miR-206 (Lin et al., 2021). In non-small cell lung cancer, Megakaryocytic leukaemia 1 (MKL1)-induced lncRNA SNHG18 encouraged metastatic growth and proliferation by sequestrating miR-211-5p, which results in the upregulation of its downstream target Bromo-domain containing protein 4 (BRD4), which is a well-known regulator for cancer development (Fan et al., 2021). LncRNA LINC00467 is another such that was previously reported to have a role in glioma progression by interfering with DNMT1 binding to p53, which is an essential component for the initiation and progression of glioma. It was found that LINC00467 promoted glioma cell proliferation and invasion by reducing the p53 expression (Zhang et al., 2020). Not limited to its activity in glioma, LINC00467 was also found to be actively involved in lung adenocarcinoma (LAD) contributing to its poor prognosis. The proliferative role of LINC00467 was further confirmed by its knockdown in LAD cell lines which resulted in inhibition of cell proliferation as well as cell apoptosis promotion via interacting with miR-4779 and miR-7978 (Chang and Yang, 2020). These findings further clue towards the already reported associative role of lncRNAs with miRNAs in cancer metastasis providing deeper insights into the complex oncogenic pathology (Ren et al., 2024; Wang and Chen, 2023). Similarly, a cytoplasmic lncRNA LINC00909 was found to inhibit SMAD4 expression at the post-transcriptional level thereby activating the MAPK/JNK signaling pathway leading to tumorigenicity and high metastasis in pancreatic cancer (Li et al., 2024).

**TABLE 5:** Role of lncRNAs in Metastasis.

Sl no.	Name of lncRNA	Type of cancer	Function	References
1	HOTAIR	Breast cancer	Modulates miR-130a-3p expression and regulates metastatic spreading via Suv39H1/AKT/mTOR axis	He et al. (2022a)
		Prostate cancer	Inhibits hepaCAM expression and promotes metastasis by activation of MAPK pathway	Li et al. (2021a)
2	MALAT1	Head and neck squamous cell carcinoma	Suppresses Von Hippel–Lindau tumor suppressor (VHL) and inhibits apoptosis	Duan et al. (2023)
3	NKILA	Oral and oesophageal squamous cell carcinoma	Inhibits phosphorylation of IκBα, thereby mitigating metastasis via downregulating NF-κB signalling	Hu et al. (2020), Lu et al. (2018)
4	NORAD	Breast cancer	Sponges miR-155-5p and increase the expression of tumour suppressor SOCS1	Liu et al. (2021c)
		Lung cancer	Acts as a ceRNA for miR-28-3p, resulting in the upregulation of oncogenic transcription factor E2F2	Tao et al. (2022)
5	SNHG3	Gastric cancer	Prevents the association of miR-139-5p to transcription factor MYB, promoting metastasis	Xie et al. (2021)
6	SNHG14	Hepatocellular carcinoma	Interacts with miR-206 and promotes upregulation of SOX9, inhibiting apoptosis and encouraging metastasis	Lin et al. (2021)
7	SNHG18	Non-small cell lung cancer	Sequestrates miR-211-5p, resulting in the upregulation of BRD4	Fan et al. (2021)
8	LINC00467	Glioma	Promotes glioma cell proliferation and invasion by interfering with DNMT1 binding to p53 and reducing p53 expression	Zhang et al. (2020)
		Lung adenocarcinoma	Interacts with miR-4779 and miR-7978, causing metastasis and proliferation	Chang and Yang (2020)
9	LINC00909	Pancreatic cancer	Inhibits SMAD4 expression, activating the MAPK/JNK signaling pathway, leading to tumorigenicity and high metastasis	Li et al. (2024)
10	LOC105369504	Colorectal cancer	Binds directly to the protein of paraspeckles compound 1 (PSPC1) and regulates its stability using the ubiquitin-proteasome pathway causing suppression of progression and metastasis of colorectal cancer	Zhan et al. (2023)
11	LIMT	Colorectal cancer	Blocks the Proliferation and Metastasis of Colorectal Cancer Cells via Regulating miR-27b-3p/HOXA10 Axis	Li and Guo (2020)
		Breast cancer	Inhibits cell proliferation and invasion through the Wnt/beta-catenin signaling pathway in breast cancer	Yuan et al. (2019)
		Gastric cancer	Regulates miR-27a-3p/TET1 axis leading to inhibition of growth and metastasis of GC cells	Guo and Li (2020)
		Hepatocellular cancer	Inhibits EGF-induced invasion and EMT.	Hu et al. (2022)
12	LncRNA-LET	Hepatocellular cancer	Inhibits NF90, which is known to promotes tumorigenicity through PI3K/Akt pathway	Yang et al. (2013)

## 5 LncRNAs in tumor-leukocyte consortium: inflammation and immune escape in the cancer microenvironment

The cellular diversity of the cancer microenvironment provides an intriguing facet of study for understanding the complex interactions occurring within the tumour itself. Among the varying heterogeneity of cells present in the cancer matrix, leucocytes play some of the most important roles associated with the regulation of the disease condition, from encouraging apoptosis and inflammation in tumour tissues, demonstrating ameliorative action on the disease to promoting angiogenesis, immune suppression and metastasis of cancer cells, contributing to cancer growth and development. Tumour-associated macrophages (TAMs) are one of the most abundant leucocytes found in the tumour microenvironment, mainly recruited in their monocyte form by the cancer cells via secretion of chemokine signals like C-C motif chemokine ligands (CCL2, CCL18, CCL20), C-X-C motif chemokine ligands (CXCL12), VEGF-A etc., commonly differentiating into two conflicting subgroups–M1 and M2 macrophages. These groups differ in morphology, phenotype, inductivity and activity. T_H_1-induced M1 macrophages promote anticancer activities through the expression of pro-inflammatory and immune-stimulatory cytokines, exhibiting phagocytosis and can be activated by pro-inflammatory signals like Interferon-γ (IFN-γ), lipopolysaccharides (LPS), TNF-α to produce reactive oxygen species (ROS) and nitric oxide (NO), stimulating apoptosis and cell death. In contrast, M2 macrophages are generally activated by T_H_2 cell responses (IL-4, IL-10 and IL-13) and show immunosuppressive activities like inhibition of T-cell responses, overexpression of scavenger receptors like CD68, CD163 and CD206, which are associated with expression of high expression of IL-10, IL-1β, VEGF, and MMPs and upregulation of NF-κβ-mediated factors that protect against apoptosis-like IL-1β, IL-6, TNF-α, CCL2, CXCL8, and CXCL10, ultimately supporting tumour progression, growth, invasion and metastasis (Xiang et al., 2021; Zhu et al., 2021). Other than TAMs, Tumour-infiltrating lymphocytes (TILs) are also present in the tumour microenvironment, comprising heterogeneous populations of CD4^+^, CD8^+^ (T cells), CD20^+^ (B cells), NK and T regulatory cells. TILs are emerging as a promising approach in cancer therapy when used in combination with conventional chemotherapeutic approaches, suppressing cancer progression through the generation of cytotoxic and cell-mediated immune responses in tumorigenic conditions and promoting ameliorative effects (Paijens et al., 2021; Liu D. et al., 2022; Kazemi et al., 2022).

The interrelation of lncRNAs with inflammation and immune response has been the topic of extensive study over the years (Walther and Schulte, 2021), and the effects of such RNA molecules on the behaviour of the TAMs in the tumorigenic microsystem provide valuable insight into the process of tumour growth progression and metastasis. Han et al., 2021 investigated the effects of lncRNA CRNDE on the progression of liver cancer. CRNDE showed significant upregulation in THP-1 cell lines when co-cultured with LC cell line H22 and overexpression of the lncRNA induced M2 polarisation in the macrophage cells via upregulation of IL4/13 stimulation, leading to expression of M2 markers such as CD163, TGFβ, IL10, CCI22, CCL22 being increased as well in the co-cultured macrophages compared to control. Similar results were observed in normal THP-1 cells induced with IL4/13 as co-culture of the induced macrophages with Human umbilical vein endothelial cells (HUVEC) cells upregulated VEGF, Notch1, Dll4 and VEGFR2 expression in HUVEC cells, promoting migration and indicating the role of M2 macrophages in the angiogenesis of cancer cells (Han et al., 2021). Intergenic non-coding RNAs LINC00665 and LINC00337 promoted the shift of macrophage polarity to M2 and enhanced tumour progression. In gastric cancer, overexpression of LINC00665 induces M2 polarisation in macrophages via targeting transcription factor BTB domain and CNC homology 1 (BACH1), boosting Wnt signalling and is connected to increased immunogenic tolerance and progression of the tumour, decreasing overall survival in cancer patients (Yang et al., 2022). LINC00337, on the other hand, showed an increased expression profile in breast cancer and was directly responsible for the increment in expression of M2 markers like IL13, CCL2 and Macrophage-colony stimulating factor (M-CSF), promoting M2 polarisation. Furthermore, such changes enhanced breast cancer cell survival, providing chemoresistance against paclitaxel and also promoting cancer progression through overexpression of EMT markers like N-cadherin and vimentin (Xing et al., 2021). LncRNA Small Nucleolar Host Gene 1 (SNHG1) promoted M2 polarisation in breast cancer-associated macrophages as evidenced by the attenuation of the macrophage RAW 264.7 activation and downregulation of M2 markers after IL4/13 stimulation in SNHG1 knockout conditions. Furthermore, SNHG1 knockdown also downregulated STAT/JAK signalling and inhibited tumorigenesis and migration of MCF-7 cells by regulating CD36 and CD206 markers (Zong et al., 2021). The decreased expression profile of TAM-associated lncRNA Growth Arrest Specific 5 (GAS5) was correlated with the negative prognosis in endometrial cancer. Overexpression of GAS5 encouraged anti-tumorigenic conditions in EC mainly in 3 ways–promoting M1 polarisation in TAMs by microRNA-21– phosphatase and tensin homolog (PTEN)–AKT axis (miR-21/PTEN/AKT axis), increasing phagocytosis and immune cell activation in cancer environment and suppressing the nuclear accumulation and phosphorylation of oncogenic yes-associated protein 1 (YAP1) in TAMs (Tu et al., 2022). In ovarian cancer, Extracellular vesicles from the TAMs support the immune escape of cancer cells via the transfer of non-coding RNA NEAT1. NEAT1 acts as a ceRNA in oncogenic cells, targeting miR-101-3p and enhancing the expression of ZEB1, resulting in the proliferation of ovarian cancer cells, tumour growth and apoptosis of CD8^+^ cells via miR-101-3p/ZEB1/PD-L1 pathway (Yin and Wang, 2023). Yao et al. demonstrated that nasopharyngeal cancer cells-associated exosomal RNA TP73-AS1 increased NPC proliferation by sponging miR-342-3p and also supported pro-tumor M2 polarisation of TAMs via transfer through exosomes as witnessed by increment of M2 markers like CD206 and MRC-2 in control macrophage cells when co-cultured with TP73-AS1 overexpressing CNE-2 cells (Yao et al., 2022). LncRNA HOTAIR, as observed by Wang et al., when transferred via exosomes from dysregulated cells of the tumour to the macrophages in the vicinity, can promote M2 polarisation in laryngeal squamous cell carcinoma as evidenced by the increased expression of CD163 and CD206 markers. Furthermore, THP-1 macrophages co-cultured with TU212 and TU177 showed increased expression of IL10, CCL18 and IL4 and have been shown to promote EMT in LSCC via modulation of PTEN/PI3K/AKT axis (Wang J. et al., 2022). In hepatocellular carcinoma, MEG3 overexpression by the LPS/IFN-γ induced M1 macrophages demonstrated antitumor effects as MEG3 expression in the cancer cells was significantly reduced and increased expression of MEG3 by the M1 macrophages suppressed metastasis and angiogenesis of HCC cells by targeting miR-145-5p and downregulating the production of disabled 2 (DAB2) protein. MEG3 also inhibited IL4/13 stimulated M2 polarisation as upregulation of MEG3 expression in M2-Bone Marrow-Derived Macrophages (M2-BMDMs) downregulated M2 markers like CD206, YM1, MRC and ARG1, ameliorating tumour proliferation in macrophage-Huh7 co-culture (Wei et al., 2023). LncRNA HIF-1A stabilising long non-coding RNA (HISLA) derived from exosomes of M2 macrophages promoted bladder cancer proliferation and migration via stabilisation of β-catenin (Guo Y. et al., 2022) and pancreatic cancer-derived exosomal lncRNA FGD5-AS1 enhanced M2 polarisation in TAMs by interacting with p300, promoting acetylation of STAT3 and activating STAT3/NF-κβ highway (He Z. et al., 2022). Silencing of lncRNA DCST1-AS1 inactivated the NF-κβ pathway, repressed M2 polarisation and inhibited the proliferation of Oral Squamous Cell Carcinoma, thereby regulating tumour growth and associated inflammation (Ai et al., 2021). NIFK-AS1 attenuated M2 polarisation in endometrial cancer-associated macrophages by sponging miR-146a, resulting in upregulation of Notch1 signalling (Zhou et al., 2018) and exosomal lncRNA RPPH1 in colorectal cancer promoted M2 polarisation, causing metastasis of cancer cells (Liang et al., 2019).

However, unlike TAMs, the association of lncRNAs with TILs has been a relatively recent topic of research. Deng et al., 2022, identified 10 TIL-related lncRNAs that are differentially expressed in patients suffering from renal cell carcinoma using various statistical analyses. Among these non-coding RNA molecules, lncRNA AC084876.1 demonstrated a significant association with the progression and prognosis of RCC. Downregulation of AC084876.1 decreased the expression of PD-L1 and TGF-β and prevented the growth and invasiveness of cancer cells (Deng et al., 2022). Tao et al. also developed a prognostic model of TIL-based lncRNA expression in breast cancer using Kaplan-Meier survival analysis, Multivariate Cox regression analysis, and receiver-operating characteristic (ROC) curves. The model successfully predicted overall survival and was able to distinguish between cold and hot tumours, establishing a crosslink between the TIL-mediated expression of lncRNAs and the progression of cancer (Tao et al., 2022).

## 6 LncRNAs as prognostic marker and therapeutic target for cancer

The involvement of lncRNAs in the interactions taking place within the oncogenic microsystem encourages their use as a therapeutic target for the attenuation of disease-associated damage. Furthermore, the prospect of lncRNA-based prognosis of cancer is a frontier that is being extensively explored nowadays. Tumor suppressor long intragenic non-coding RNA p53-induced transcript (LINC-PINT) has a role in a variety of cancers and malignant processes. Nasopharyngeal cancer, renal carcinoma, non-small cell lung cancer, glioblastoma, thyroid cancer, retinoblastoma, ovarian cancer, breast cancer, oesophageal squamous cell carcinoma, osteosarcoma, melanoma, and gastric cancer are among those in which LINC-PINT is downregulated. Furthermore, decreasing LINC-PINT expression indicates advanced clinical tumour stages and a bad prognosis. Reduced LINC-PINT expression promoted interaction with BRAF-activated non-coding RNA/mitogen-activated protein kinase, preventing tumour migration, invasion and proliferative growth, sensitised triple-negative breast cancer cells (TNBC) to chemotherapeutics and sponged miR-374a-5p, suppressing the invasiveness of ovarian cancer (He T. et al., 2021). Upregulated lncRNA DLX6-AS1 indicate poor prognosis in bladder cancer, breast cancer, glioma, ovarian cancer, cervical cancer, osteosarcoma, and lung cancer, among others and can be a candidate for therapeutic targeting (Zheng et al., 2021). MALAT1 and HOTAIR showed significant fluctuation in their expression under various cancer types, influencing inflammation, autophagy, migration, metastasis and therapeutic sensitisation, highlighting their potential for acting as a biomarker and therapeutic candidate (Goyal et al., 2021; Raju et al., 2023; Xin et al., 2021). However, use of lncRNAs as prognostic markers in cancer is quite challenging as tissue specific expression of lncRNAs can significantly alter the diagnostic pattern. Furthermore, some lncRNAs have shown contradictory expression as well as mode of action in different cancer types, adding an extra layer of complexity in prognosis.

Therapeutics modulating lncRNA expression in cancer has been one of the more recent topics of research. Several synthetic and natural compounds have shown the ability to regulate the synthesis of these non-coding RNA molecules and elicited profound effects on the cellular signalling and events going on in the complex cancer microenvironment. Curcumin, a polyphenolic compound mainly isolated from *Curcuma longa* and used for its antioxidant and anti-inflammatory effects, demonstrated inhibitory effects on the EMT of breast cancer cells *in vivo*. Curcumin modulated the expression of the lncRNA H19, which exhibited pro-EMT effect and downregulation of the RNA molecule by curcumin treatment halted migration and invasiveness of Tamoxifen-resistant MCF-7 cells, normalising E-cadherin expression while decreasing N-cadherin production (Cai et al., 2021). Furthermore, Khan et al. used a combination of Curcumin along with N-n-butyl haloperidol iodide (F2) in hepatocellular carcinoma. The combination (F2C) alleviated the proliferative malignancy of the cancer cells by downregulating enhancer of zest homolog 2 (EZH2) transcription and protein expression, resulting in decreased migration, tri-methylation of histone H3 at lysine 27 (H3K27me3) and long non-coding RNA H19 expression (Khan et al., 2021). LncRNA PVT1 displayed upregulation in cisplatin-resistant ovarian cancer cells and promoted proliferation and migration by increased PD-L1 expression. Atezolizumab treatment and knockdown of PVT1 expression showed a synergistic effect on the growth of ovarian cancer and promoted apoptosis of A2780cis cells by downregulating the JAK/STAT3/PD-L1 axis (Chen et al., 2021c). Quercetin, a flavonoid known for its antioxidant, anti-inflammatory and anticancer activity, was studied for its regulatory capability on the lncRNA transcriptome by Chai et al., 2021. They found that quercetin worked against lncRNA SNHG7 as treatment of the natural compound-induced apoptosis and arrested proliferation and metastasis in non-small-cell lung carcinoma cells via upregulation of miR-34a-5p, which repressed SNHG7 expression (Chai et al., 2021). Metformin, a biguanide drug known for its antidiabetic activity, attenuated lncRNA H19 expression in breast cancer cells. Downregulation of H19 by metformin promoted ferroptosis in MCF-7 cells by increasing ROS production and decreasing reduced Glutathione (GSH) levels in the cancer cells. Furthermore, metformin also inhibited autophagy in the treated cells, suppressing beclin-1 and LC3 production, all of which were reversed by the overexpression of H19 (Chen et al., 2022). Astragaloside IV, one of the active ingredients of the herb *Astragalus membranaceus*, is a cyclobutane-type triterpene glycoside that demonstrated various pharmacological activities, including anticancer, immune-regulatory, antioxidant, neuroprotective, and cardioprotective abilities. Astragaloside IV has been shown to quell the spreading of breast cancer via the promotion of lncRNA Thyrotropin Releasing Hormone Degrading Enzyme Antisense RNA 1 (TRHDE-AS1). Astragaloside IV-mediated upregulation of TRHDE-AS1 was shown to be correlated with the decreased expression of key protein markers of tumour growth and metastasis like Proliferating Cell Nuclear Antigen (PCNA), MMP9 and MMP7, ameliorating breast cancer proliferation and migration (Hu et al., 2021). Ginsenoside compound K, a major deglycosylated metabolite of ginseng found in organs or blood after oral ingestion of PPD ginsenosides in the human gastrointestinal (GI) tract, usually known for its apoptosis-inducing effects of cancer cells, inhibited the malignancy of renal cell carcinoma by targeting lncRNA THOR (Testis-associated Highly-conserved Oncogenic long non-coding RNA). THOR was significantly upregulated in RCC cells *in vitro*, and compound K treatment repressed the expression of THOR, promoting apoptosis and ROS production. Silencing of the lncRNA exhibited similar effects as compound K treatment, establishing the connection of THOR with the oncogenesis of RCC and compound K as a possible therapeutic option for treatment (Chen S. et al., 2021). Shi et al. investigated the mitigatory action of gallic acid on hepatocellular carcinoma. Gallic acid regulated the expression of MALAT1, which was implicated with the EMT and tumorigenesis of HCC, and also extinguished the migratory status of cancer cells by downregulating the MALAT1/Wnt/β-catenin axis (Shi et al., 2021). Ketamine, a dissociative anaesthetic mainly used for pain management in cancer, promoted anti-proliferative status in cancer cells by modulating the production of lncRNA PVT1. Ketamine dampened PVT1 expression in SKOV3 and OVCAR3 ovarian cancer cells *in-vitro* and suppressed cell growth by binding to EZH2, a subunit of polycomb repressive complex 2, resulting in the increased expression of cyclin-dependent kinase inhibitor p57 (Li et al., 2021b) and also promoted ferroptosis in liver cancer cells by targeting PVT1, causing overexpression of the downstream target of the lncRNA, miR-214-3p. MiR-214-3p, in turn, competitively binds to GPX4, inducing iron-mediated apoptosis via PVT1/miR-214-3p/GPX4 axis in liver cancer (He GN. et al., 2021).

## 7 Discussion and future prospective

LncRNAs regulate key cellular mechanisms in various organs of our body through their diverse interactome and have a major hand in the initiation, regulation and progression of diseases related to dysregulation of cellular functions. In the cancer microenvironment, the multidimensional interactome of lncRNAs has been shown to regulate key events of cancer development, mainly via modulating fundamental cellular processes like apoptosis, autophagy and migration. Additionally, the lncRNA-based interactions between the immune cells, i.e., macrophages and lymphocytes, with the cancer cells in the oncogenic microhabitat shape the inflammatory landscape for the growth, immune escape of tumour cells and progression of cancer. Alteration in the sequence and structure of lncRNA can lead to the alteration in regulatory properties of the molecule, and disease-associated mutations have also demonstrated significant prevalence among individuals in the diseased population (Castellanos-Rubio and Ghosh, 2019). This makes lncRNAs an important target as well as an attractive candidate for therapeutic purposes. Several synthetic and natural compounds have been found to regulate lncRNA expression in several cancer types *in vivo*, inhibiting cancer growth and metastasis [163-1172, (Han et al., 2022)]. However, the focus of lncRNA-targeted therapeutics is still largely fixed on the anticancer frontier and should be expanded to encompass inflammatory and autoimmune diseases as well. CRISPR-mediated knockdown of lncRNA expression can be a frontier in assessing the functional aspect of non-coding RNA molecules. However, the genomic complexity of lncRNAs, the presence of multiple introns and the relative genomic overlapping of lncRNAs with the nearby protein-coding genes pose a challenge to the CRISPR/Cas9-based genome editing approach (Rabaan et al., 2023; Lyu et al., 2023). Since the expression and interactome of a particular lncRNA can vary between different organ systems of the body, there is still a lot to be known about the involvement of lncRNAs in disease pathways, and a lot of research still needs to be done. Furthermore, several lncRNAs are differentially expressed in the samples of diseased individuals. So, the use of lncRNA as a prognostic marker for early diagnosis of the disease is also a potential area of study, and significant work needs to be done here as well.
